# Amyloid tumor of the breast

**DOI:** 10.1186/s40792-019-0591-z

**Published:** 2019-02-19

**Authors:** Makiko Mori, Haruru Kotani, Masataka Sawaki, Masaya Hattori, Akiyo Yoshimura, Naomi Gondo, Yayoi Adachi, Ayumi Kataoka, Kayoko Sugino, Nanae Horisawa, Mitsuo Terada, Yuri Ozaki, Hiroji Iwata

**Affiliations:** Department of Breast Oncology, Aichi Cancer Center, 1-1 Kanokoden, Chikusa-ku, Nagoya, 464-8681 Japan

**Keywords:** Breast tumor, Amyloid tumor, Amyloidosis, Sjogren syndrome

## Abstract

**Background:**

Amyloid tumor of the breast is a rare disease, which was first reported in 1973. To date, only six cases have been reported in Japan.

**Case presentation:**

A 45-year-old woman who had a medical history of Sjogren’s syndrome presented with a lump of 3 cm in diameter on the outer side of the right breast. Mammography showed no abnormality. Ultrasonography showed a well-defined and rough hypoechoic mass of 32 mm in diameter at the site of the lump. With suspicion of breast cancer, an ultrasound-guided vacuum-assisted breast biopsy was performed. For pathological diagnosis, hematoxylin and eosin staining showed deposits of nonstructural substances in the interstitium. The specimen stained red with Congo red staining and showed green birefringence under a polarizing microscope. Thus, the mass was diagnosed as an amyloid tumor. Since the patient had Sjogren’s syndrome, it was considered a breast finding of autoimmune disease. We considered further therapy to be unnecessary, and annual follow-up was recommended.

**Conclusions:**

We diagnosed the mass as an amyloid tumor by an ultrasound-guided vacuum-assisted breast biopsy without resection. The patient had no systemic symptoms suspected systemic amyloidosis, and we diagnosed localized amyloidosis. An amyloid tumor of the breast may show findings suggestive of breast cancer. Pathological diagnosis before surgery is important to avoid excessive invasion. If deposits of nonstructural substances are observed by hematoxylin and eosin staining, Congo red staining should be added.

## Background

Amyloid tumor of the breast, first reported in 1973 [1], is a rare disease, with only six cases [2–7] reported in Japan to date.

Amyloidosis is defined as a disease that causes abnormalities in organs due to extracellular deposition of fibrous abnormal proteins called amyloid [8]. It is divided into systemic amyloidosis in which amyloid deposits form in organs throughout the body, and localized amyloidosis which is limited to an individual organ [8]. Systemic amyloidosis causes a variety of symptoms, such as fatigue, weight loss, anemia, cardiac symptoms (congestive heart failure, arrhythmia), renal symptoms (nephrotic syndrome, kidney failure), gastrointestinal symptoms (malabsorption syndrome, macroglossia, hepatomegaly, splenomegaly), neurological symptoms (polyneuropathy, carpal tunnel syndrome, orthostatic hypotension, constipation, diarrhea, dysuria), and bleeding symptoms [8]. Examinations used to check for systemic amyloidosis include electrocardiography, echocardiography, blood analysis (renal dysfunction, M protein, free light chain, autoimmune antibody, chronic inflammatory findings), urine analysis (Bence-Jones protein), nerve conduction test, bone marrow biopsy, and biopsy of sites suspected of amyloid deposition [8]. The diagnosis of amyloidosis is confirmed by Congo red staining which stains amyloid red, and the stained amyloid also shows green birefringence under a polarizing microscope [8].

A report of 15 patients with amyloid tumor of the breast at the Mayo Clinic showed that amyloid tumor of the breast, when a manifestation of systemic amyloidosis, is mostly found as a late presentation, and none of the patients with a localized amyloid tumor of the breast developed systemic amyloidosis [9].

## Case presentation

A 45-year-old woman originally visited a different hospital because of a focal asymmetric density of the left breast identified by screening mammography. She had a medical history of Sjogren’s syndrome. Ultrasonography showed no abnormality in the left breast, whereas an indistinct hypoechoic mass of 25 mm in diameter was detected in the outer side of the right breast. Although cytology of the right breast mass indicated no malignant feature, she came to our hospital for further examinations.

**Fig. 1:**
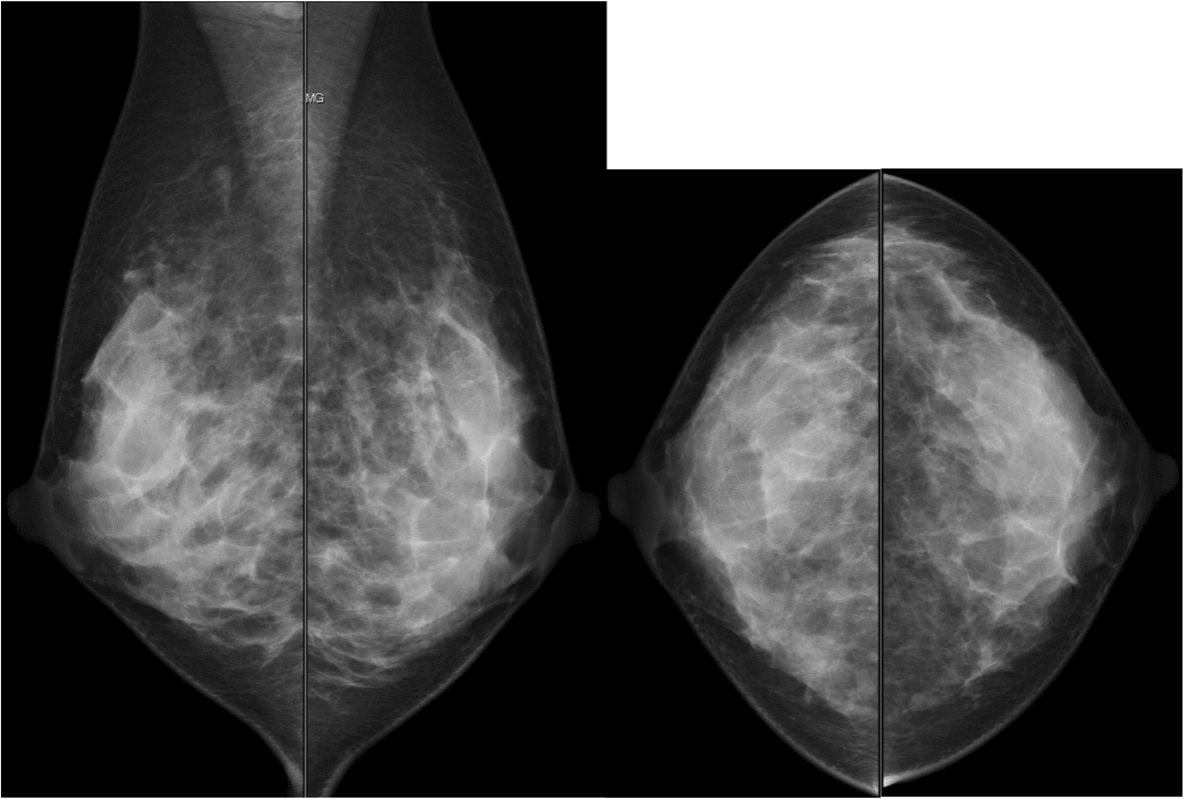
Mammogram findings. Mammography showed no abnormality

**Fig. 2:**
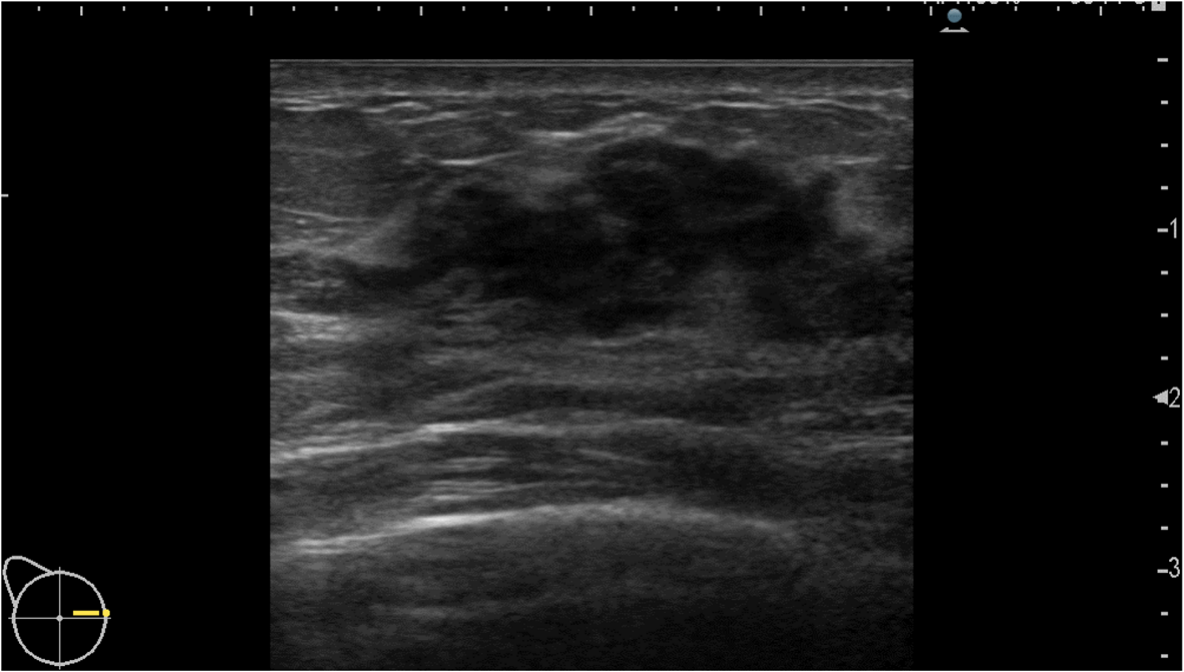
Ultrasonographic findings. Ultrasonography showed a well-defined and rough hypoechoic mass of 32 mm in diameter at the site of the lump

A lump of 3 cm in diameter was palpable on the outer side of the right breast. Mammography at our hospital showed no abnormality (Fig. 1). Ultrasonography showed a well-defined and rough hypoechoic mass of 32 mm in diameter at the site of the lump (Fig. 2). With suspicion of breast cancer, an ultrasound-guided vacuum-assisted breast biopsy was performed.

**Fig. 3:**
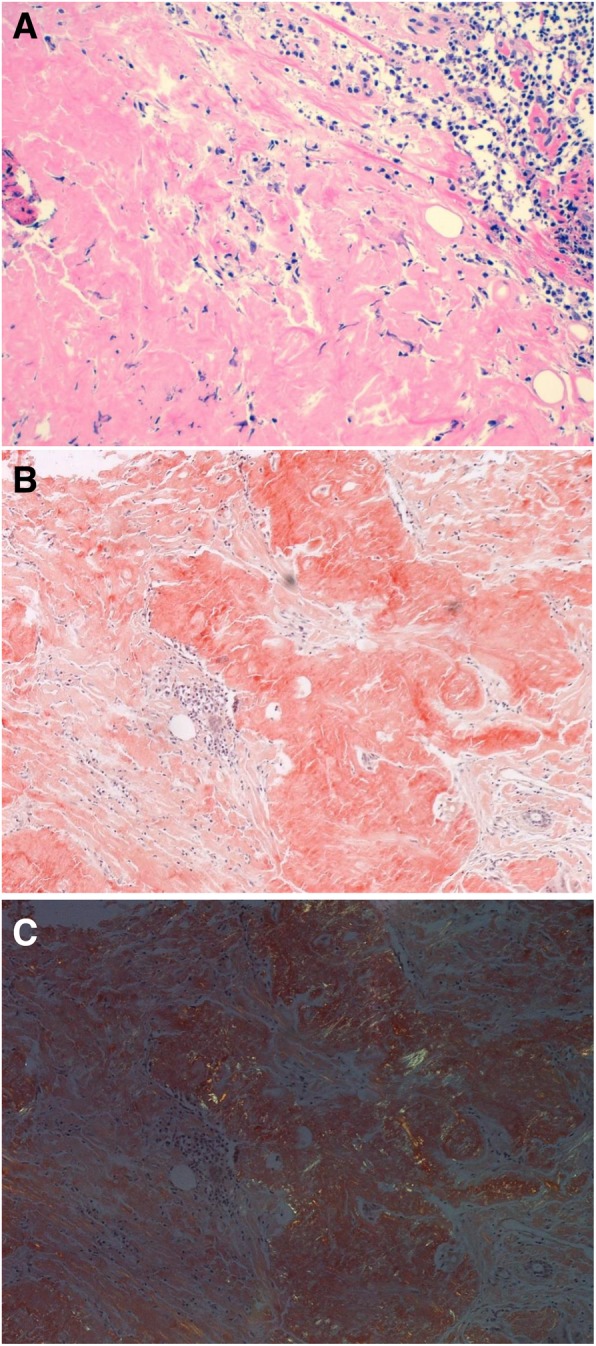
Microscopic observations. Hematoxylin and eosin staining showed deposits of nonstructural substances in the interstitium (**a**). The specimen stained red with Congo red staining (**b**), and the Congo red staining showed green birefringence under a polarizing microscope (**c**)

For pathological diagnosis, hematoxylin and eosin staining showed deposits of nonstructural substances in the interstitium (Fig. 3a). The specimen was positively stained by Congo red (Fig. 3b) and showed green birefringence under a polarizing microscope (Fig. 3c). Thus, the mass was diagnosed as an amyloid tumor. She had no systemic symptoms suggestive of systemic amyloidosis. We considered further therapy to be unnecessary, and annual follow-up was recommended.

## Conclusions

Amyloid tumor of the breast is a rare disease. To date, only six cases [2–7] have been reported in Japan (Table 1). All were women, with a median age of 67.5 years. Findings may be suggestive of breast cancer, such as microcalcifications identified by mammography or an indistinct hypoechoic mass by ultrasonography. Excisional biopsy was performed in five of the six patients, and resection for confirmation was carried out in the sixth patient. Two patients had medical histories of autoimmune disease.

**Table 1:** Amyloid tumor of the breast: reported cases in Japan

Author/year	Age/sex	MMG	US	Treatment	Medical history
Yokoo 1998	76F	Mass with micro calc.	–	Excisional biopsy	–
Honda 2000	52F	–	Hypoechoic mass	Excisional biopsy	none
Hukushima 2002	59F	–	Indistinct mass	Excisional biopsy	SLE, hemodialysis
Hosoi 2008	79F	None	Mass	Excisional biopsy	Sjogren’s syndrome
Ito 2011	57F	–	Well-defined and smooth hypoechoic mass	Excisional biopsy	None
Tsuji 2016	77F	FAD	Hypoechoic area	CNB: amyloid →resection	None
Our case 2018	45F	No abnormality	Well-defined and rough hypoechoic mass	MMT: amyloid →follow-up	Sjogren’s syndrome

All were women, and median age was 67.5 years. Findings may be suggestive of breast cancer, such as microcalcification by mammography or an indistinct hypoechoic mass by ultrasonography. Excisional biopsy was performed in five of the six cases, and resection for confirmation was performed in the other case. Two cases had medical histories of autoimmune disease

Between 1998 and 2018, only 65 patients with amyloid tumor of the breast [2–7, 9–43] have been reported worldwide including Japan. Nine patients were diagnosed with systemic amyloidosis. Five of those had already been diagnosed with systemic amyloidosis before the diagnosis of amyloid tumor of the breast. Three patients had some systemic symptoms associated with systemic amyloidosis. The remaining patient had received hemodialysis for 20 years and was diagnosed with systemic amyloidosis secondary to hemodialysis. However, we found no details regarding whether she had systemic symptoms suspected of systemic amyloidosis. We found that most patients diagnosed with systemic amyloidosis had systemic symptoms.

In our case, we were able to diagnose the mass as an amyloid tumor by an ultrasound-guided vacuum-assisted breast biopsy without resection. The patient had no systemic symptoms indicative of systemic amyloidosis, and therefore, we considered examination for systemic amyloidosis to be unnecessary and diagnosed localized amyloidosis. If the patient develops systemic symptoms, she should be checked for systemic amyloidosis.

Since the findings of amyloid tumor of the breast may be confused with breast cancer, pathological diagnosis before surgery is important to avoid excessive invasion and unnecessary surgery. If deposits of nonstructural substances are observed by hematoxylin and eosin staining, Congo red staining should be added for confirmation.
